# The diagnostic yield of inferior petrosal sinus sampling in Cushing syndrome in the era of ovine CRH shortage

**DOI:** 10.1007/s00701-024-06058-2

**Published:** 2024-04-03

**Authors:** Lukas Andereggen, Jan Gralla, Emanuel Christ

**Affiliations:** 1 Department of Neurosurgery, Kantonsspital Aarau, Aarau, Switzerland; 2 Faculty of Medicine, University of Bern, Bern, Switzerland; 3 Department of Diagnostic and Interventional Neuroradiology, Inselspital Bern, Bern University Hospital and University of Bern, Bern, Switzerland; 4 Department of Endocrinology, Diabetology and Metabolism, University Hospital of Basel, Basel, Switzerland; 5 Department of Clinical Research, University of Basel, Basel, Switzerland

**Keywords:** Cushing’s syndrome, oCRH stimulation, Drug shortages, Petrosal sinus sampling

## Abstract

**Purpose:**

The ovine corticotropin-releasing hormone (oCRH) stimulation test has been routinely used in the diagnostic work-up of ACTH-dependent Cushing syndrome (CS). With oCRH currently being out-of-stock in Europe, we aimed at evaluating the diagnostic performance of inferior petrosal sinus sampling (IPSS) without oCRH stimulation.

**Methods:**

We compared the values of 40 patients with ACTH-dependent CS and negative MRI findings in whom ACTH was measured before and after oCRH stimulation.

**Results:**

The ratio of central-to-peripheral ACTH measurement (IPS:P) before the combined 3, 5, and 10 min of oCRH stimulation yielded diminished sensitivity (85% vs. 97%), alongside markedly decreased specificity (57% vs. 71%), as well as reduced positive and negative predictive values (90% vs. 94% and 44% vs. 83%), respectively.

**Conclusions:**

With the current drug shortages in Europe, ACTH measurements without oCRH stimulation in IPSS cannot be recommended. Thus, we call for desmopressin or the commercially available human CRH as a potential alternative in the confirmation of ACTH excess by IPSS in equivocal MRI findings.

## Introduction

Inferior petrosal sinus sampling (IPSS) is an established method to identify the correct source of ACTH-dependent Cushing’s syndrome (CS) in patients with inconclusive imaging [14, 20]. Thereby, ovine corticotropin-releasing hormone (oCRH) stimulation is used to increase the sensitivity and specificity of IPSS in distinguishing Cushing’s disease (CD) from ectopic ACTH syndrome (EAS) [1, 22]. With the current drug shortages in Europe and oCRH being out-of-stock, we aimed at evaluating if inferior petrosal sinus sampling (IPSS) without oCRH stimulation can still be recommended to correctly localize the ACTH source.

## Material and methods

Retrospective cohort study including patients with ACTH-dependent CS and equivocal responses to dynamic testing (i.e., ovine corticotropin-releasing hormone [oCRH] stimulation and/or high-dose dexamethasone suppression) with missing evidence of a microadenoma on the 1.5 Tesla MRI. For the latter, a standardized protocol of PD/T2-weighted whole-brain study with unenhanced, contrast-enhanced, dynamic contrast-enhanced, and post-contrast-enhanced overlapping studies in the axial, sagittal, and coronal planes over the sellar region was used [1, 4]. For the IPSS procedure, a Hi-Flow Tracker 18 (Renegade Hi-Flow Microcatheter; Boston Scientific Target, Fremont, CA) catheter was used to bilaterally cannulate the IPS, and, if not possible, the venous outflow was recorded based upon an internal carotid artery injection. Simultaneous samples were obtained from the left and right IPS, and the peripheral venous blood both before, and at 3, 5, and 10 min after oCRH (1 μg/kg body weight) stimulation for the measurements of ACTH concentrations [4]. A central-to-peripheral (C:P) gradient ≥ 2.0 before and/or ≥ 3.0 after oCRH stimulation pointed out a central ACTH source [22], and lateralization with an intersinus gradient ≥ 1.4 [21]. A transnasal transsphenoidal surgical (TTS) approach to the pituitary gland was used [3, 5–7, 18, 19]. In case of negative intraoperative adenoma identification, the lateral third of the pituitary gland was resected on the side predicted by IPSS [2]. The accuracy of IPSS in the detection of an adenoma was based on histopathological confirmation of an ACTH-secreting adenoma [30]. As for the latter, the paraffin sections were stained for hematoxylin, reticulin, and ACTH and assess for ACTH hyperplasia or positive Crooke’s hyalinization as an indirect marker of hypercortisolism in the case no adenoma could be verified [23].

### Statistical analyses

Data were analyzed using IBM SPSS statistical software (V29.0 Software, IBM Corp., New York, NY, USA). Continuous variables were examined for homogeneity of variance and are expressed as mean ± standard deviation (SD) unless otherwise noted. Sensitivity, specificity, positive predictive value, and negative predictive value were calculated before and after oCRH stimulation.

## Results

A total of 33 (82%) women and 7 (18%) men had ACTH samples measured before and 3, 5, and 10 min after oCRH stimulation. Mean age was 45 ± 15 (range, 7–71) years. No mortality and minimal morbidity were associated with IPPS and TSS. Namely, one patient suffered from nausea potentially related to the contrast agent applied by IPSS, and one patient from postoperative transient diabetes insipidus.

A C:P gradient ≥ 2.0 was observed in 30 out of 40 patients (75%) before oCRH stimulation, and a gradient ≥ 3.0 was noted in 36 out of 40 patients (90%) after oCRH stimulation. The sensitivity and specificity of BIPSS in predicting the correct ACTH source, as confirmed by histology, were found to be 28/33 (85%) and 4/7 (57%) before oCRH stimulation, and 32/33 (97%) and 5/7 (71%) after oCRH stimulation, respectively. Consequently, the C:P ACTH measurement ratio before and after oCRH stimulation yielded positive and negative predictive values of 28/31 (90%) and 4/9 (44%) before vs. 32/34 (94%) and 5/6 (83%) after oCRH, respectively (Table 1).

**Table 1:** Comparison between baseline and post-oCRH test characteristics for bilateral inferior petrosal sinus sampling

ACTH measurements w/o and w oCRH stimulation	Baseline (none)	3 min	5 min	10 min	After (mean)
Sens., TP/(TP + FN) (%)	28/33 (85)	32/33 (97)	30/33 (91)	31/33 (94)	32/33 (97)
Spec., TN/(FP + TN) (%)	4/7 (57)	5/7 (71)	4/7 (57)	4/7 (57)	5/7 (71)
PPV, TP/(TP + FP) (%)	28/31 (90)	32/34 (94)	30/33 (91)	31/34 (91)	32/34 (94)
NPV, TN/(FN + TN) (%)	4/9 (44)	5/6 (83)	4/7 (57)	4/6 (67)	5/6 (83)

*ACTH*, adrenocorticotropin; *FN*, false negative; *FP*, false positive; *NPV*, negative predictive value; *oCRH*, ovine corticotropin-releasing hormone; *PPV*, positive predictive value; *Sens.*, sensitivity; *Spec.*, specificity; *TN*, true negative; *TP*, true positive

Concerning the lateralization data of adenoma in patients with Cushing’s disease (CD), the normalized ACTH values (represented as side-to-side gradients) showed no significant differences between patients with symmetric outflow (11.9 ± 3.3) and those with asymmetric outflow at baseline (16.4 ± 6.0; *p* = 0.22). However, they exhibited significant disparities after oCRH stimulation (symmetric: 5.5 ± 2.3 versus asymmetric: 35.7 ± 22.5; *p* = 0.01). As a result, the prediction of adenoma presence was marginally better when venous outflow was symmetric (100%) compared to asymmetric (83%), though the distinction was not statistically significant (*p* = 0.53).

## Discussion

The current data suggest that the sensitivity of ACTH measurements without oCRH stimulation is diminished, and alongside the poor specificity of unstimulated IPSS, it prohibits their application during oCRH shortages in European countries.

The current gold standard in the work-up for CD in elusive MRI findings is the application of IPSS with oCRH stimulation [28]. IPSS can accurately differentiate a central pituitary lesion from a peripheral ectopic ACTH source [11], yet stimulation with oCRH is imperative to increase its correctness [25]. Namely, with oCRH stimulation, results of IPSS have been associated to robustly confirm or exclude the diagnosis of CD with an 80–100% sensitivity and over 95% specificity [12, 16, 17, 22, 27]. Thereby, an asymmetric venous outflow does not appear to significantly affect the precision with which the adenoma side can be predicted [4], although results after oCRH stimulation show significant differences with larger numerical variations. Nonetheless, perhaps owing to the small patient cohort, it does not seem to influence clinical outcomes, at least to a noticeable extent. Very recently, an European multicenter study used human CRH (hCRH) as an alternative stimulant showing a sensitivity of 81.1% (95% CI 72.1–87.8) and a specificity of 85.7% (95% CI 56.2–97.5) applying the conventional cut-off values of ≥ 3 for post-hCRH stimulation, and a sensitivity 94.3% (95% CI 87.6–97.7) and specificity 85.7% (95% 56.1–97.5) using an newly calculated optimized cut-off values of ≥ 2.1 post-hCRH [16]. Alternatively, desmopressin might be used instead given its availability and easy applicability. Desmopressin is a synthetic analogue of human vasopressin that has been used to replace oCRH to stimulate ACTH secretion for IPSS. Namely, desmopression has been related to an increased secretion of ACTH in CD patients but not in those with EAS given the overexpression of V2 desmopressin receptors in corticotrope adenoma cells only [13]. Nevertheless, no firm recommendation can be currently drawn, as most studies using desmopression are limited by small sample sizes [28]. Other promising results with regard to correct adenoma localization have been attributed to molecular imaging with (1) the use of ^11^C-methionine (MET)-positron emission tomography (PET) fused with MRI [9, 10] and (2) the administration of ^68^ Ga-CRH PET-CT[29]. While both imaging techniques are based on small number of patients, the availability of the tracers and the radiological infrastructure limits the use of these interesting methods. On the other hand, better visualization and thus higher detection rates of pituitary lesions at a 7.0 Tesla MRI vs. 1.5 Telsa MRI in CD patients has been described [15]. While a 7.0 Tesla MRI might become a solid adjunct in the non-invasive workup of CD patients [24], its current availability and significance remain elusive.

Taken together, despite the fact that alternative methods with interesting results were reported, the data are based on small sample sizes and availability of these methods are restricted. It is, therefore, probably too early to draw firm conclusion and we still rely on a stimulated IPSS to correctly localize the ACTH source in ACTH dependent CD. Desmopressin or hCRH alike might become a valuable alternative to oCRH in the diagnostic work-up of CD patients with equivocal MRI findings.

## Conclusions

Our results indicate that the specificity of IPSS without oCRH stimulation in indicating the correct ACTH location is poor and cannot be recommended. At this time, we thus call for the use of the wider available desmopressin or hCRH as an alternative until the European drug shortage of oCRH becomes solved.

## Study limitations

The present study has certainly some limitations; besides its retrospective nature, the number of patients who underwent bilateral IPSS is relatively small but results are based on data collected over a decade, with homogeneity in terms of indications, treatment, and follow-up. It is worth noting that in 7/40 (17.5%) patients, the type of drainage according to the classification described by Shiu et al. [26] and Benndorf et al. [8] could not be determined due to missing angiographic data that was already time-barred.

## Data Availability

The authors agree to share data upon request.

## Code availability

N/A.
